# Discrimination of consonants in quiet and in noise in Mandarin-speaking children with normal hearing

**DOI:** 10.1371/journal.pone.0283198

**Published:** 2023-03-21

**Authors:** Lena L. N. Wong, Shufeng Zhu, Yuan Chen, Xinxin Li, Wing M. C. Chan

**Affiliations:** 1 Faculty of Education, The University of Hong Kong, Pokfulam, Hong Kong SAR, China; 2 Department of Special Education and Counselling, The Education University of Hong Kong, Hong Kong SAR, China; Education University of Hong Kong, HONG KONG

## Abstract

**Objective:**

Given the critical role of consonants in speech perception and the lack of knowledge on consonant perception in noise in Mandarin-speaking children, the current study aimed to investigate Mandarin consonant discrimination in normal-hearing children, in relation to the effects of age and signal-to-noise ratios (S/N).

**Design:**

A discrimination task consisting of 33 minimal pairs in monosyllabic words was designed to explore the development of consonant discrimination in five test conditions: 0, -5, -10, -15 dB S/Ns, and quiet.

**Study sample:**

Forty Mandarin-speaking, normal-hearing children aged from 4;0 to 8;9 in one-year-age increment were recruited and their performance was compared to 10 adult listeners.

**Results:**

A significant main effect of age, test conditions, and an interaction effect between these variables was noted. Consonant discrimination in quiet and in noise improved as children became older. Consonants that were difficult to discriminate in quiet and in noise were mainly velar contrasts. Noise seemed to have less effect on the discrimination of affricates and fricatives, and plosives appeared to be to be more difficult to discriminate in noise than in quiet. Place contrasts between alveolar and palato-alveolar consonants were difficult in quiet.

**Conclusions:**

The findings were the first to reveal typical perceptual development of Mandarin consonant discrimination in children and can serve as a reference for comparison with children with disordered perceptual development, such as those with hearing loss.

## 1. Introduction

With the extensive adoption of universal neonatal hearing screening in mainland China, an increasing number of Mandarin-speaking children are being diagnosed with hearing loss (HL) and subsequently fitted with hearing aids or cochlear implants (CIs) [1]. Previous studies have evaluated the speech perception of Mandarin-speaking children with HL as an outcome from auditory intervention in quiet and in noise [2–6], and consonant perception exhibits a moderate relationship with sentence perception in noise [2, 6]. However, the development of speech perception in Mandarin-speaking children with normal hearing (NH), which could serve as a reference for comparison with children with HL [7], has yet to be examined in quiet and in noise. Hence, the primary aim of the current study was to investigate the development of consonant perception in Mandarin-speaking children with NH at different signal-to-noise ratios (S/N).

### 1.1 Mandarin consonants

A Mandarin syllable can be maximally structured as consonant–vowel–consonant. While vowels are an essential component of Mandarin syllable structures, consonants are optional. There are 21 Mandarin consonants that can be placed in a syllable-initial position, contrasted by place and manner of articulation, aspiration, and voicing [8] (Table 1). The Mandarin consonant inventory differs from English in that fewer phonemes are contrasted by voicing (i.e., there are eight pairs of voicing contrasts in English but one pair in Mandarin), but aspiration is a more frequent phonemic feature (i.e., there are no aspiration contrasts in English but six pairs in Mandarin). Furthermore, there are three palato-alveolar phonemes in Mandarin, which are rare in other languages [9]. There are only two Mandarin consonants (/n/ and /ŋ/) that can be placed in syllable-final position. Due to the limited number of contrasts, the perception of final consonants will not be explored in the current study.

**Table 1:** Mandarin syllable-initial consonants cited (Peng et al., 2004).

Manner of articulation		Place of Articulation and Voicing (-/+)												
		Bilabial		Labiodental		Alveolar		Palato-alveolar		Palatal			Velar	
	Aspiration	-	+	-	+	-	+	-	+	-	+		-	+
Plosive	-	p				t							k	
	+	p^h^				t^h^							k^h^	
Nasal			m				n							
Fricative				f		s		ʂ	ʐ	ɕ			x	
Affricate	-					ts		tʂ		tɕ				
	+					ts^h^		tʂ^h^		tɕ^h^				
Lateral							l							

*Note*.“-” represents unaspirated or voiceless consonant and “+” represents aspirated or voiced consonant.

The ability to discriminate varies with the types of consonants. When given a two-alternative forced-choice identification task in quiet, Mandarin perception in children with CIs and hearing aids is significantly poorer than those with NH [4]. Among the six consonant contrast subcategories evaluated in Liu et al. [4], children with HL had more difficulties identifying consonants contrasted by fricative/nonfricative, same place of articulation but different manners, and same manner of articulation but different places and retroflex/nonretroflex. No significant difference was observed between children with NH and HL when consonants were contrasted by voicing and aspiration, although results from individual consonant contrasts were not reported. Cabrera et al. [10] examined discrimination between manner and place of articulation contrasts in quiet for a group of children using CIs and a group of children with NH. The results showed children with CIs had significantly more difficulty in discriminating consonant place contrasts compared to other contrasts. No significant difference in discrimination was observed between consonant place contrasts and other contrasts in children with NH.

### 1.2 Factors affecting consonant discrimination

#### 1.2.1 Age

In NH children, consonant perception improves over time, and performance becomes more adult-like as they reach 12–14 years of age [11]. For example, using a multitalker babble noise at +13 dB SNR, Johnson [12] found that children 10 to 11 years of age were significantly poorer in identifying the place of articulation and voicing, compared to adults. Using the Mandarin version of Hearing in Noise Test, Chen and Wong [13] reported sentence reception thresholds in speech-spectrum shaped noise (SSN) noise approximated adult-like performance at age 12–14.

Given the effects of age, the task to assess consonant perception must be age-appropriate. In the current study, a discrimination task (i.e., judging whether two sounds are the same or different) with items within the vocabulary repertoire of children as young as 4 years of age, was used to examine the development of consonant discrimination in children in the second and third year of kindergarten (i.e., K2 and K3) and first and second year of primary school (i.e., P1 and P2). A discrimination task was reportedly more appropriate for preschool children or children with hearing impairment [6]. Grouping based on year of education, instead of chronological age, was due to the following reasons. First, with children entering kindergarten or primary schools, parents and clinicians were more concerned about whether the consonant discrimination ability of children with HL was comparable to that of children with NH in the same class or grade, instead of “aged peers”. Second, children in the same grade are more likely to develop cognitively at about a similar rate as they share similar language experience in the classroom [14]. This may lead to reduced variations in consonant discrimination compared to those of the same chronological age.

#### 1.2.2 Effects of noise

Listening conditions are another factor influencing consonant perception. As noise is commonly present in daily listening, it is essential that we understand consonant discrimination under different levels of noise [15, 16]. Cooke [17] showed that consonants were not perceived with the same accuracy in noise by adults with NH, and found /t/, /g/, and /tʃ/ were easier to perceive, while /b/ and /v/ were more difficult to identify in most SSN maskers at -6 to 8 dB S/N, whereas Johnson [12] reported that voicing was the easiest to identify compared to place or manner of articulation features in a multitalker babble at +13 dB S/N. In contrast, Zhu et al. [6] reported low accuracy in Mandarin consonant discrimination in noise, compared to in quiet, but no significant difference in the discrimination of 16 Mandarin consonant minimal pairs in SSN at -10, -5, 0. 5, and 10 dB S/N. As there are few studies that examined consonant discrimination in noise, in particular in Mandarin, the current study employed a full range of consonant minimal pairs to examine the effects of age and noise on children with NH.

## 2. Method

### 2.1 Participants

Forty native Mandarin-speaking children with NH were recruited from mainstream kindergartens and primary schools in Shenzhen, Guangdong Province, China. To control for influences from exposure to other languages or dialects, parents completed a questionnaire to indicate the children’s language exposure. All participants had been exposed to Mandarin in most daily situations. Their parents/caregivers were also native Mandarin speakers. As teachers in Shenzhen schools are required to pass a Mandarin/Putonghua Proficiency Test, we expect that these children had been exposed to good quality Mandarin on a daily basis. According to their grade level, the children were divided into four age groups in one-year increments: K2, K3, P1, and P2. Age and gender distributions are shown in Table 2. Parents and teachers reported no otologic disorders, hearing loss, or intellectual disabilities. To compare performance with adults, ten native Mandarin-speaking adults with NH served as a control group. All participants had normal hearing sensitivity, passing a hearing screening at 20 dB HL bilaterally at 500, 1000, 2000, and 4000 Hz. The study was approved by the Human Research Ethics Committee, the University of Hong Kong. A written consent form was obtained from parents before the testing.

**Table 2:** Age and gender distributions of children in the present study (N = 40).

Group	Age range	Mean age (year; month)	No. of children		
			Male	Female	Total
P2	7;8–8;9	8;0 (96.3 months)	3	7	10
P1	6;5–7;6	7;1 (85.4 months)	5	5	10
K3	5;3–6;2	5;8 (68.0 months)	8	2	10
K2	4;0–4;8	4;4 (51.7 months)	4	6	10

*Note*: K2 means the second year of kindergarten; K3 means the third year of kindergarten; P1 means the first year of primary school; P2 means the second year of primary school.

### 2.2 Stimuli

A total of 33 consonant minimal pairs were created by contrasting different phonemic features among all 21 Mandarin initial consonants (Appendix 1). Consonants in the minimal pairs were contrasted by one phonemic feature only, with the same vowel and the same tone. The words were selected from the Dictionary of Modern Chinese and from textbooks for primary school students in year one (P1) and year two (P2) in China. To ensure age appropriateness, 10 speech-training schoolteachers completed a 5-point rating scale [1–5] for 124 words selected in the initial stage, with “1” indicating the words were inappropriate for evaluating children aged 4 and above, and “5” indicating the words were most appropriate. Words that were rated “4” and above were selected.

The 66 selected word stimuli were spoken by a male, native standard Mandarin speaker. The consonants were recorded in a sound treated room at a sampling rate of 48 kHz, using an AKG C3000B microphone placed 20 cm away from the speaker. The speaker was instructed to speak the target words in a natural manner. Each pair was recorded three times. Two native Mandarin-speaking adults were instructed to select the most naturally spoken pair among the three. If the two adults did not agree with their choices, a third native Mandarin-speaking adult listened to the selected stimuli and selected the most naturally spoken stimuli.

The Matlab program was used to calculate and equate the root-mean-square (RMS) amplitude of each recording, and the contrastive pair was spliced with a 1-second silence in between the words. A stationary SSN masker was created by filtering white noise using a finite impulse response filter based on the average spectrum from all test stimuli, and then the SSN was scaled and leveled. A custom software program was developed to administer the test and record the test results. A practice test was also created.

### 2.3 Procedure

A discrimination task was employed because it is appropriate for evaluating young children, in particular, those with poor hearing. Zhu et al. [6] proved the successful use of a discrimination task consisting of 16 consonant minimal pairs in children with severe to profound hearing loss.

The test was performed in a quiet room with noise level below 40 dBA, measured with a Sauter SU 130 sound level meter. Test materials were presented on an 11-inch touch screen laptop via a pair of Creative Gigaworks T40 2.0 loudspeakers, placed at 0 (signal) and 180 (noise) degrees azimuths, 1 meter away from the center of the head of participants. A SSN noise was used for calibration and to mask sentences in noisy conditions. In the quiet condition, the stimuli were fixed at 65dB SPL. For noisy conditions, the noise level varied to achieve 0, -5, -10, and -15 dB S/N while the stimuli were fixed at 65dB SPL.

In each condition, each minimal pair was presented four times (e.g., /t-t^h^/, /t^h^-t/, /t-t/, and /t^h^-t^h^/), so as to balance the chance of “same” and “different” answers, resulting in a total of 132 test items (= 33 consonant minimal pairs ⊆ 4 test sets). The software randomized the order of the items. The answer was recorded by the software when the child pressed the screen. Each test condition took about 15 to 25 minutes to complete, resulting in a total of 75 to 125 minutes of test time carried out in one to two sessions occurring not more than one week apart. Total test time depended on the amount of break required by the participants.

Two light-up buttons on the touch screen were used to attract the attention of participants. After the presentation of each pair of stimuli, participants were required to identify whether the two stimuli were the same or different by tapping one of the two corresponding symbols on the touch screen. A third button was used to indicate a nil response when the participants could not perceive the stimuli. Three children in K2 had difficulty responding by tapping the touch screen; therefore, head nodding and shaking or verbal responses to indicate their answer were accepted instead. Other than these three children, all others were able to complete the discrimination task independently, without further instruction or prompting from the tester.

## 3. Results

### 3.1 Consonant discrimination under five test conditions

**Fig 1:**
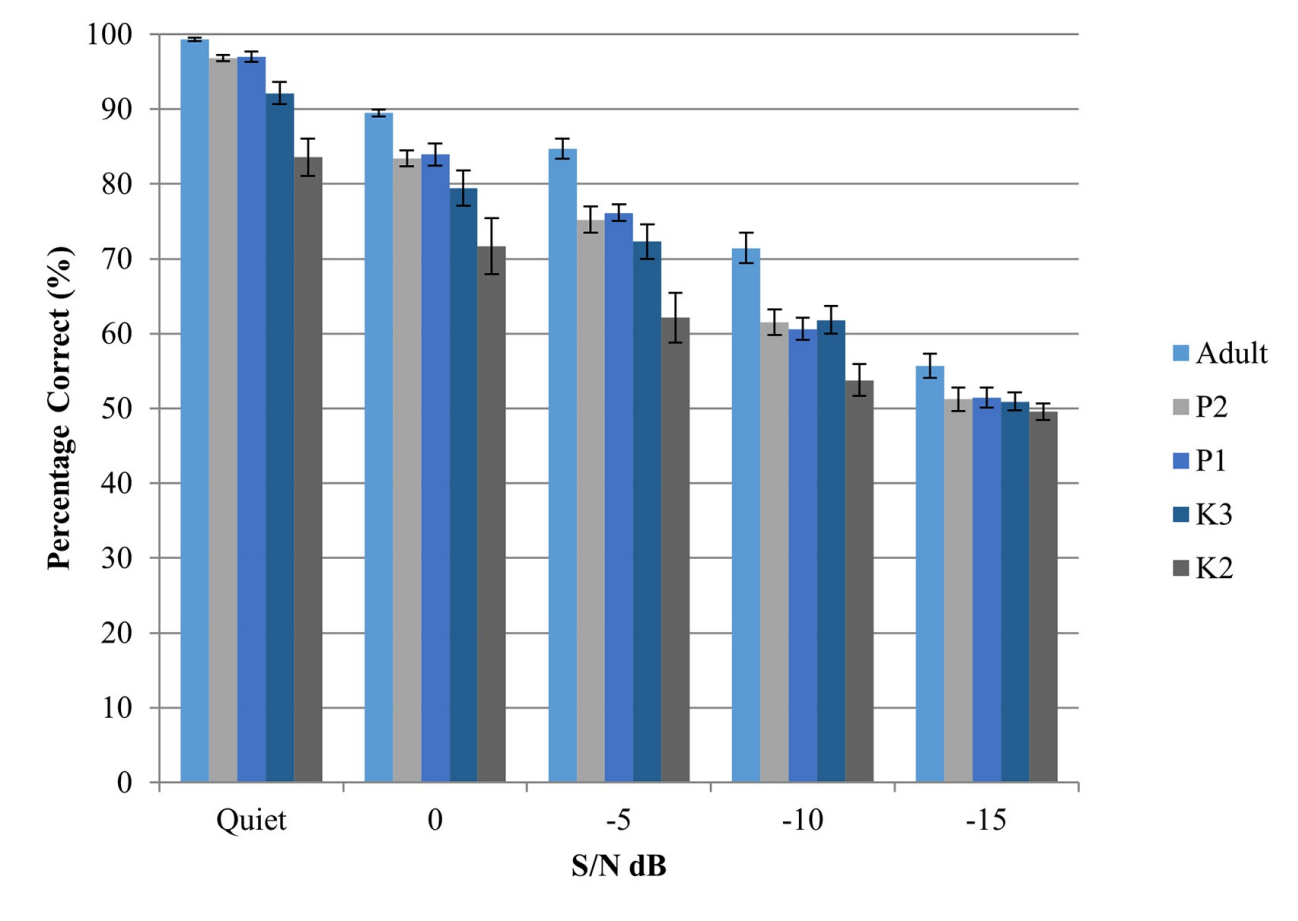
Consonant discrimination obtained in the five test conditions across the five age groups.

The overall mean discrimination scores obtained in the five test conditions are shown in Fig 1 and Table 3. A two-way mixed effects ANOVA (with test conditions as the within-subject variable and age as the between-subject variable) was further conducted to examine the effects of age and test condition on consonant discrimination. The results show significant main effects of age, *F*(4, 45) = 18.54, *p* < .001, η^2^ = 0.62 and test conditions, *F*(3.2, 142.1) = 731.67, *p* < .001, η^2^ = 0.94, on consonant discrimination.

**Table 3:** Accuracy (%) of consonant discrimination in the five test conditions across the five age groups.

Age groups	Test conditions (S/N dB)									
	Quiet		0		-5		-10		-15	
	*M*	*SE*	*M*	*SE*	*M*	*SE*	*M*	*SE*	*M*	*SE*
Adults	99.32	(0.21)	89.47	(0.46)	84.70	(1.30)	71.44	(2.07)	55.68	(1.66)
P2	96.82	(0.43)	83.41	(1.10)	75.23	(1.74)	61.52	(1.68)	51.21	(1.59)
P1	96.97	(0.67)	83.94	(1.48)	76.14	(1.08)	60.61	(1.46)	51.44	(1.34)
K3	92.12	(1.49)	79.47	(2.36)	72.27	(2.29)	61.82	(1.87)	50.91	(1.23)
K2	83.56	(2.52)	71.67	(3.74)	62.12	(3.30)	53.79	(2.12)	49.55	(1.15)

In addition, significant interactions between age and test condition were found, *F*(12.6,142.1) = 3.85, *p* < .001, η^2^ = 0.26. This indicates that age had different effects on consonant discrimination, depending on the test conditions. A post hoc one-way ANOVA was conducted to further examine the effects of age on consonant discrimination under each test condition. A significant main effect of age was noted across all test conditions (all *ps* <. 05). The results from post hoc comparisons with Bonferroni adjustment are listed in Table 4, suggesting that the K2 group performed significantly worse than the other groups in most of the test conditions, and K3 participants performed worse than adults most of the time. However, there are no significant differences among P2, P1, K3, and K2 participants at -10 and -15 S/N, suggesting floor effects. More specifically, at -10 dB S/N, discrimination scores across the four age groups ranged from 53.79% to 61.52%, which is near chance level. In the -15 dB S/N condition, performance was at chance level even for adults, suggesting that this condition is probably too difficult to be useful for distinguishing performance across listeners. Therefore, results of the -10 dB S/N and -15 dB S/N conditions were excluded from further analysis. In addition, results obtained at 0 dB S/N and -5 dB S/N were averaged to represent performance in noise, for ease of analysis.

**Table 4:** Post-hoc comparisons examining the effects of age across all test conditions.

Age groups		Test conditions (S/N dB)				
		Quiet	0	-5	-10	-15
		*p value*				
Adults	P2	1	0.526	0.026*	0.005*	0.296
	P1	1	0.759	0.060	0.002*	0.385
	K3	0.005*	0.020*	0.001*	0.007*	0.206
	K2	0*	0.000*	0.000*	0.000*	0.035*
P2	P1	1	1	1	1	1
	K3	0.187	1	1	1	1
	K2	0.000*	0.004*	0.001*	0.051	1
P1	K3	0.154	1	1	1	1
	K2	0*	0.002*	0.000*	0.127	1
K3	K2	0.001*	0.138	0.013*	0.037*	1

Note:

* indicates significant difference in consonant discrimination score with Bonferroni adjustment.

### 3.2 Discrimination of consonant minimal pairs in four categories in quiet and noise

**Fig 2:**
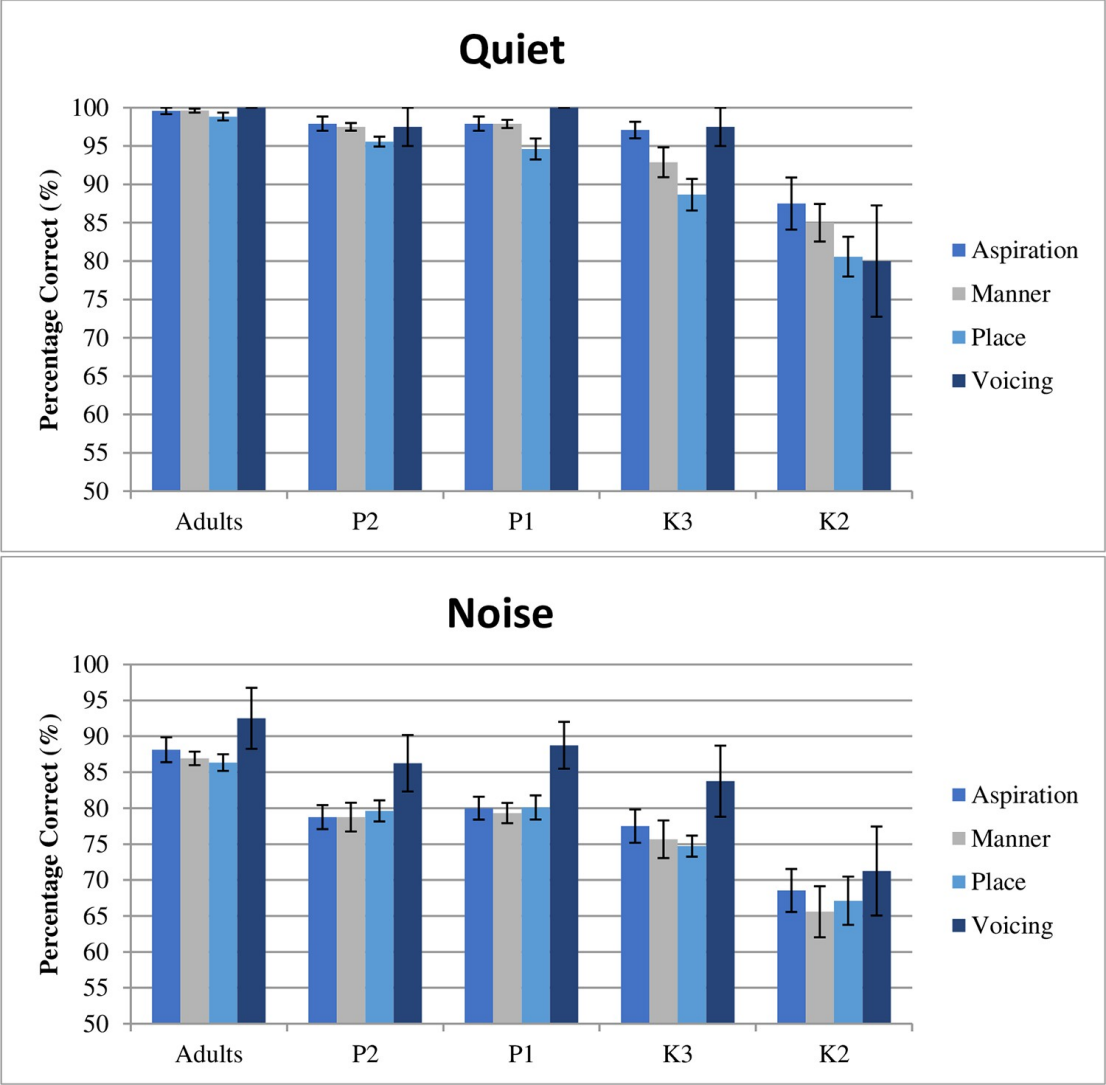
Discrimination of consonant minimal pairs in four subcategories in quiet and in noise (results were averaged from 0 and -5 dB S/N) (N = 40).

The discrimination scores of consonant pairs contrasted by aspiration, manner of articulation, place of articulation, and voicing are shown in Fig 2.

A three-way mixed effects ANOVA, with age as the between-subject variable and test condition and contrast category as within-subject variables, was conducted to examine the effects of age, test condition, and contrast category on consonant discrimination ability. A significant main effect of age (*F*(4, 45) = 16.26, *p* < .001, η^2^ = 0.59), test condition (*F*(1, 45) = 331.06, *p* < .001, η^2^ = 0.88), and contrast category (*F*(1.5, 65.8) = 11.08, *p* < .001, η^2^ = 0.20) were noted. There were no significant triple interaction effects (age x test condition x contrast category) (*F*(6.0, 67.3) = 0.29, *p* > .05, η^2^ = 0.03). However, there were significant interactions between contrast category and test conditions (*F*(1.5, 67.3) = 6.12, *p* < .01, η^2^ = 0.12). Post hoc pairwise comparisons with Bonferroni adjustment showed the following findings: 1) adults, P1, P2, and K3 children performed significantly better than K2 children; 2) the contrast of aspiration and the contrast of manner were significantly better discriminated than place of articulation in the quiet condition. However, children and adults demonstrated similar performance in the discrimination of minimal pairs contrasted by aspiration and place and manner of articulation in noise; and 3) consonant minimal pairs contrasted by voicing were significantly more accurately discriminated compared to the other three categories in noise.

### 3.3 Discrimination of consonant minimal pairs in quiet and noise

Discrimination scores for the 33 consonant minimal pairs across age groups in quiet and in noise are listed in Appendix 2 and 3, respectively. The minimal pairs that scored one standard deviation lower than the overall mean of the 33 consonant pairs were considered difficult, and those that scored one standard deviation above the overall mean were considered easy. This criterion was used in Zhu et al. [6]. Furthermore, a paired samples t-test with Bonferroni adjustment was conducted to compare the performance of each minimal pair obtained in the quiet and noisy conditions.

#### 3.3.1. Easy Pairs in quiet and in noisy conditions

**Fig 3:**
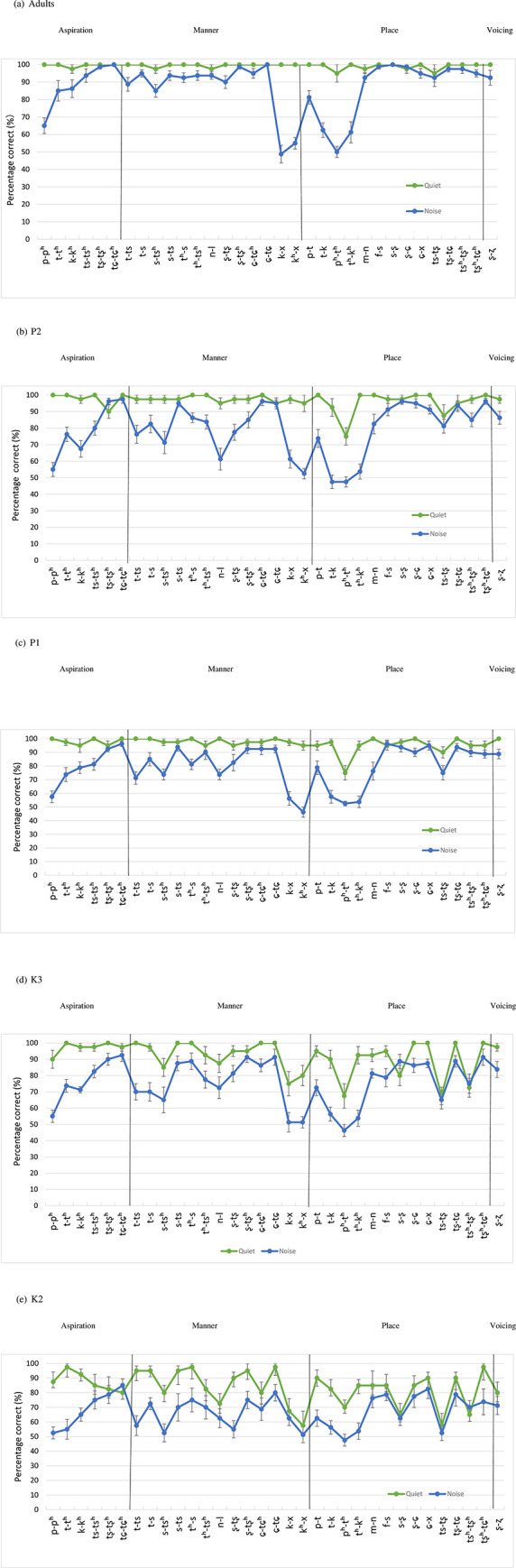
Discrimination of 33 consonant minimal pairs in quiet and in noise across the five age groups: (a) adults; (b) P2; (c) P1; (d) K3; (e) K2.

In quiet, ceiling effect was observed for all age groups (see Fig 3 and Appendix 2 for detail scores). The adults achieved over 95.0% accuracy with all 33 minimal pairs, and the two older age groups (P1 and P2) achieved 95.0% accuracy for approximately 90% of the minimal pairs. However, children in the K3 group were able to discriminate 63.6% (21 out of 33) of the minimal pairs with the same accuracy level (95%), and the K2 children were only able to reach this criterion with 24.2% (8 out of 33) of the contrastive pairs. In general, younger children were able to discriminate fewer consonant pairs with 95.0% or above accuracy In addition, there were no significantly easy pairs for Adults, P2, P1 and K3, possibly due to ceiling effects. However, /tʂ-tʂ^h^/, /tʂ^h^-tɕ^h^/, /ɕ-tɕ/, and /t-t^h^/ were relatively easy for K2 children.

In noise, large variations in performance were noted across age groups (Fig 3 and Appendix 3), with older children performing better. Across age groups, the minimal pair /tɕ-tɕ^h^/ was easiest to discriminate; /tʂ-tʂ^h^/, /tʂ^h^-tɕ^h^/, /ɕ-tɕ/, and /ɕ-x/ were also relatively easy when +1 *SD* from the overall mean was used as the cutoff criterion. Overall, contrastive pairs composed of affricates and fricatives posed less difficulty in noisy conditions across all ages (Fig 3).

#### 3.3.2 Difficult Pairs in quiet and in noisy conditions

In quiet, the ability to discriminate difficult contrastive pairs improved with participant age (Appendix 2). The K2 and K3 groups had difficulty with similar contrastive pairs in quiet, including /k^h^-x, k-x, p^h^-t^h^, ts-tʂ, s-ʂ, ts^h^-tʂ^h^/, although K3 children were more accurate compared to the K2 ones. Children above age 6 performed even better on the same contrasts, although their performance was still below that of adults.

In noise, a developmental trend in consonant discrimination was not evident, as /t^h^-k^h^/, /p-p^h^/, /k^h^-x/, /k-x/, /p^h^-t^h^/ were found to be difficult across all age groups, including adults (Appendix 3). As shown in Fig 3, the plosives and velar sounds were difficult regardless of age. Although fricatives and affricates were relatively easy for most children, when a cutoff criterion of 1 *SD* lower than overall mean was used, they were still difficult for the youngest children.

While some contrastive pairs are more difficult to discriminate in noisy conditions than in quiet (see Appendix 2 & 3), the contrastive pair of aspirated plosives /p^h^-t^h^/ was more difficult across age groups in both quiet and in noise, followed by the contrasts of velar sounds /k^h^-x, k-x/. In addition, the discrimination of place contrasts of alveolar and palato-alveolar sounds (/s-ʂ, ts-tʂ, ts^h^-tʂ^h^/) were particularly more difficult compared to other alveolar contrasts.

**Fig 4:**
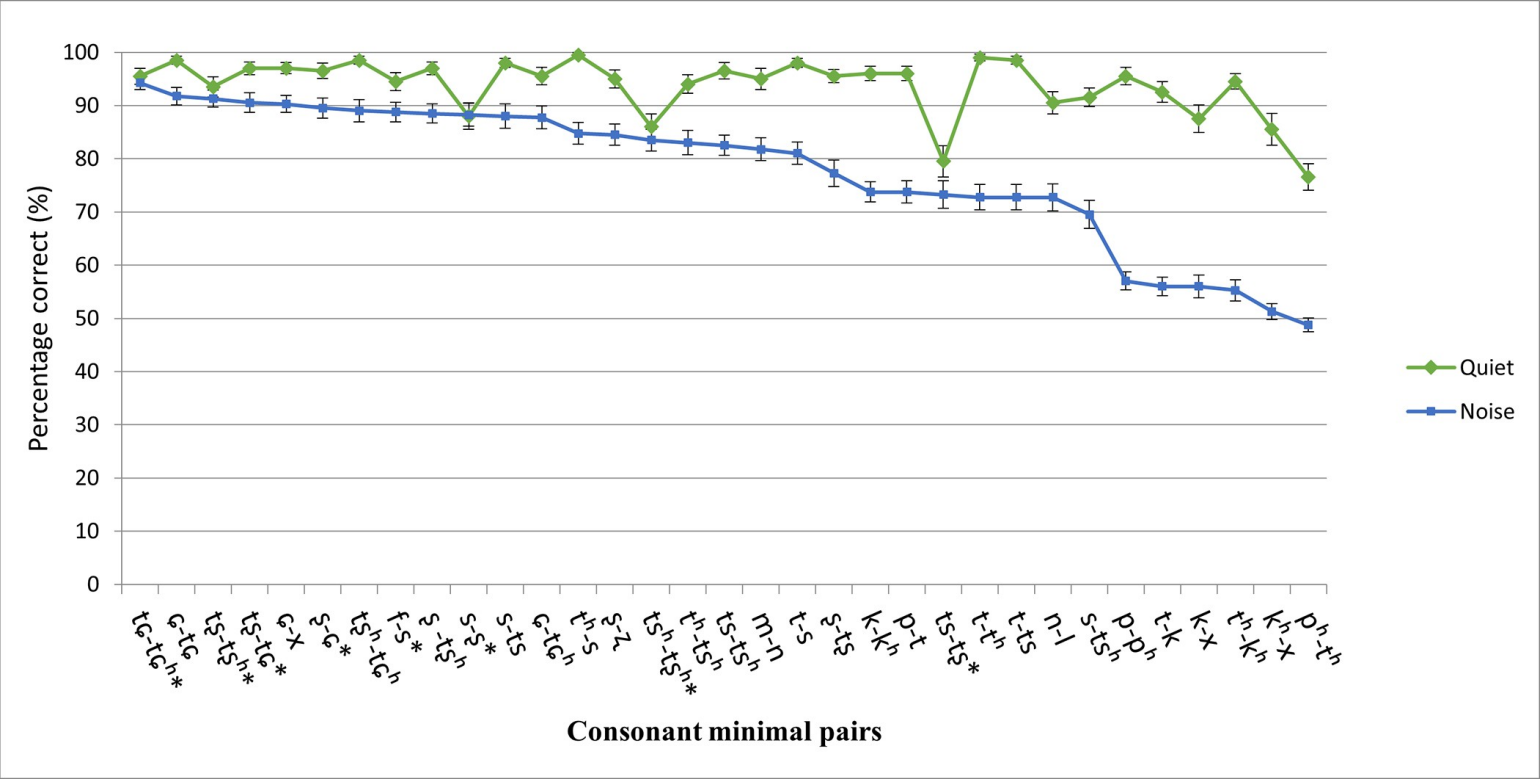
Discrimination of 33 consonant minimal pairs in quiet and in noise across all listeners. Consonant pairs were arranged according to performance obtained in noise (*N* = 40). *indicates no significant difference between performance in quiet and in noise.

However, some easy consonant pairs (mainly affricates and fricatives) were not affected by noise (e.g., /tɕ-tɕ^h^, tʂ-tʂ^h^, f-s, tʂ-tɕ, ʂ-ɕ/) (Fig 4). Some pairs were easy to discriminate only in quiet but not in noise, in particular, plosives contrasted by aspiration (e.g., /p-p^h^/, /k-k^h^/, and /t-t^h^/); furthermore, some plosives contrasted by place of articulation and some manner contrasts were also relatively easy in quiet but quite difficult in noise (e.g., /p-t/, /t^h^-k^h^/, /t-k/, and /t-ts/).

#### 3.3.3 Order of discrimination acquisition in quiet and in noisy conditions

To determine the age of acquisition, we referred to the criterion used in speech sound production literature [18–20], where a sound is considered acquired/mastered when 90% of the children in a particular age group are able to produce it. In the current study, each contrastive pair was tested four times, and to avoid occasional errors in the discrimination task, a contrastive pair was considered learned by a child when it was discriminated correctly in at least three out of four (75%) opportunities. Table 5 summarizes the developmental trend of Mandarin consonant discrimination with reference to the criterion that 90% of participants were able to achieve a score of 75% or above on each consonant. As a result, 54.5% (18 out of 33) of the contrastive pairs was found to be acquired by K2 children under quiet listening conditions, while no contrastive pair was acquired by the same group of children in noisy conditions. Among all pairs, /pʰ-tʰ/ was the only pair that could not be discriminated by 90% of children in quiet conditions, including those in P2. However, about 45% (15 out of 33) of the contrastive pairs were not mastered by P2 children in noisy conditions. The discrimination of the velar contrasts /k-x, kʰ-x/ and place contrasts of alveolar and palato-alveolar sounds, /ts-tʂ, tsʰ-tʂʰ, s-ʂ/ was acquired by P1 children but posed great difficulty for K2 and K3 children. In general, consonant discrimination in quiet was near completion by the time children reached P1. Discrimination was much harder in noisy conditions, and seven contrastive pairs could not be discriminated by adults. These include /p-t, pʰ-tʰ, t-k, tʰ-kʰ, p-pʰ, k-x, and kʰ-x/, suggesting that the difficulty in discriminating speech sounds in noisy conditions is governed not only by developmental changes but also by acoustic factors.

**Table 5:** Consonants that could be discriminated by more than 90% of children with over 75% correct discrimination in quiet and in noise (results were averaged from 0 and -5 dB S/N).

	90% of children with over	
	Quiet	Noise
Adults	All	All but p-t, pʰ-tʰ, t-k, tʰ-kʰ, p-pʰ, k-x, kʰ-x
P2	All but p^h^-t^h^	ts-ts^h^, ʈʂ- ʈʂ^h^, tɕ- tɕʰ, s-ts, t^h^-s, ʂ -ʈʂ^h^, ɕ-tɕʰ, ɕ-tɕ, m-n, s-ʂ, ʂ-ɕ, ɕ-x, tʂ-tɕ, tʂ^h^-tɕ^h^, ʂ- ʐ, t^h^-ts^h^, f-s, tʰ-tsʰ
P1	t-t^h^, k-k^h^, ts-ts^h^, t-ts, t-s, s-ts^h^, s-ts, t^h^-s, ʂ-ʈʂ, ʂ -ʈʂ^h^, ɕ-tɕ, p-t, t^h^-k^h^, m-n, f-s, ɕ-x, tʂ-tɕ, tʂ^h^-tɕ^h^, p-p^h^, ʈʂ- ʈʂ^h^, tɕ- tɕʰ t^h^-ts^h^, n-l, ɕ-tɕʰ, t-k, ʂ-ɕ, ʂ- ʐ, **k-x, k**^**h**^**-x, s-ʂ, ts-tʂ, ts**^**h**^**-tʂ**^**h**^ (i.e., all but p^h^-t^h^)	ts-ts^h^, ʈʂ- ʈʂ^h^, tɕ- tɕʰ, s-ts, t^h^-s, ʂ -ʈʂ^h^, ɕ-tɕʰ, ɕ-tɕ, m-n, s-ʂ, ʂ-ɕ, ɕ-x, tʂ-tɕ, tʂ^h^-tɕ^h^, ʂ- ʐ, **t**^**h**^**-ts**^**h**^**, f-s, tʰ-tsʰ**
K3	t-t^h^, k-k^h^, ts-ts^h^, t-ts, t-s, s-ts^h^, s-ts, t^h^-s, ʂ-ʈʂ, ʂ -ʈʂ^h^, ɕ-tɕ, p-t, t^h^-k^h^, m-n, f-s, ɕ-x, tʂ-tɕ, tʂ^h^-tɕ^h^**, p-p**^**h**^**, ʈʂ- ʈʂ**^**h**^**, tɕ- tɕʰ** **t**^**h**^**-ts**^**h**^**, n-l, ɕ-tɕʰ, t-k, ʂ-ɕ, ʂ- ʐ**	**ts-ts** ^**h**^ **, ʈʂ- ʈʂ** ^**h**^ **, tɕ- tɕʰ,** **s-ts, t** ^**h**^ **-s, ʂ -ʈʂ** ^**h**^ **, ɕ-tɕʰ, ɕ-tɕ, m-n, s-ʂ, ʂ-ɕ, ɕ-x, tʂ-tɕ, tʂ** ^**h**^ **-tɕ** ^**h**^ **, ʂ- ʐ**
K2	t-t^h^, k-k^h^, ts-ts^h^, t-ts, t-s, s-ts^h^, s-ts, t^h^-s, ʂ-ʈʂ, ʂ -ʈʂ^h^, ɕ-tɕ, p-t, t^h^-k^h^, m-n, f-s, ɕ-x, tʂ-tɕ, tʂ^h^-tɕ^h^	None

Note.

* Each contrastive pair was tested four times and those that could be discriminated at least three (75%) out of the four contrastive pairs are listed. Consonants were regarded as having been acquired by participants in an age group if at least 90% of them were able to achieve a discrimination score of at least 75% accuracy. Older children were able to discriminate more minimal pairs and the additions for each group as compared to the younger age group have been bolded.

## 4. Discussion

In the current study, the development of consonant perception in both quiet and noisy conditions was examined using a discrimination task consisting of 33 consonant minimal pairs. The children had no difficulty in completing the task, implying the task was appropriate.

### 4.1 Effects of age on consonant discrimination

The present study revealed a significant effect of age on the ability to discriminate consonants, indicating that the impact of age should be considered when evaluating if a child with HL is developing consonant discrimination on par with same-aged peers or when tracking outcomes with hearing device longitudinally. The maturation effect observed in the present study has also been reported in previous studies on child speech production development [11, 13, 21, 22]. However, this is the first study that reports on consonant discrimination among Mandarin-speaking children with NH. Except for the discrimination of a few contrasts, ceiling effects were observed for a large number of consonant pairs in noisy conditions across age groups. That is, discrimination of /p^h^-t^h^/ was slightly more difficult for children in P1 and P2, and /k^h^-x, k-x, p^h^-t^h^, ts-tʂ, s-ʂ, and ts^h^-tʂ^h^/ posed great difficulty for children in K2 and K3, but other contrastive pairs were well discriminated. Furthermore, consonant discrimination in quiet listening conditions is near completion by about age 7 (see Table 4), which is similar to reports on English-speaking children [23]. The trajectory of development slows but continues with ongoing refinement after age 8, as children in the oldest group were still unable to achieve adult-like performance under quiet conditions. Previous studies have also showed that perceptual discrimination of English phonemic contrasts for children aged from 6:0 to 12:6 was less accurate than for adults [24]. This maturation effect could be attributed to the following two reasons. First, the lack of accumulated experience with the phonological system and less experience with lexical cues may contribute to a child’s immature perception of consonant contrasts [25]. Liu et al. [26] found that the ability to discriminate specific speech cues in Mandarin consonants, measured using mismatch responses, continues to improve in preschool and school years. This suggests that young children had less precise neural representation of consonants and thus were less efficient in using lexical cues for consonant perception. In addition, maturational effect may be associated with age-related changes in auditory system [13, 22]. Sanes and Woolley [27] reported that high-level auditory processing abilities (e.g., amplitude modulation detection, frequency discrimination, auditory perceptual learning) are not as good as adults until children reach 10–14 years of age.

### 4.2 Effects of noise on consonant discrimination

Across most consonants, discrimination was more difficult in noise than it was in quiet. The negative impact of noise declines from pre-school to early school years, and the declining effects of masking with age are expected until the late teenage years or even adulthood [12, 28]. Younger children may not be able to ignore irrelevant sound features in noisy listening conditions. However, certain consonants were difficult to discriminate in noisy conditions regardless of age, suggesting that these difficulties are perceptual rather than developmental. The evaluation of consonant discrimination in noisy conditions successfully demonstrated further maturation effects that were not observed in the quiet condition. Thus, evaluation of consonant discrimination should be extended to listening in noisy conditions, particularly when performance in quiet already reaches maximum.

Previous studies have also revealed a significant effect of noise on consonant perception in children with HL [6] and adults with NH [29]. The discrimination of fricatives and affricates was an exception. That is, the consonant pairs (e.g., /tʂ-tʂ^h^, tɕ-tɕ^h^, ʂ-ɕ, tʂ-tɕ, f-s/) were found to be easy to discriminate both in quiet and in noise. Wright [30] pointed out that sibilant fricatives are the most resistant to masking due to their higher intensity of noise and longer onset release bursts compared to plosives and non-sibilant fricatives. This was also confirmed by Meyer et al. [31], in which English phoneme recognition was evaluated. In the current study, results from three contrasting sibilant fricatives /ɕ, s, ʂ/ in Mandarin provided further empirical evidence for this claim. However, bilabial and dental plosives contrasted by aspiration were not easy to discriminate in noise, despite high discriminability in quiet. Wong [32] also found affricates easier to perceive in noise compared to plosives in a study of Cantonese-speaking NH adults. Again, the longer duration of frication and the greater intensity in affricates compared to plosives may account for the ease in discrimination in noisy conditions [30, 33]. In addition, discrimination involving place contrasts of alveolar and palato-alveolar sounds (/s-ʂ, ts-tʂ, ts^h^-tʂ^h^/) was particularly more difficult in quiet. However, the discrimination of these consonant minimal pairs was not affected by noise (i.e., discrimination scores obtained in quiet and in noise did not differ significantly). These findings provide further evidence for the robustness of the acoustic information associated with fricatives and affricates in noise.

Regardless of listening conditions, the plosives contrasted by place of articulation /p^h^-t^h^/ and velar contrasts /k^h^-x, k-x/ were difficult to discriminate. Although Zhu et al. [6] also found the discrimination of /p^h^-t^h^/ difficult and attributed this to difficulty in discriminating place contrasts, other place contrasts were not as difficult for Mandarin-speaking children. Regarding the discrimination of /k^h^-x, k-x/, the low-frequency nature of velar sounds might have led to the difficulty, as low-frequency cues are easily masked by noise [34] and discrimination of lower frequency sounds does not mature until later, at about ten to eleven years old [35]. Similarly, brief low-frequency bursts in plosives serve as main cues for their distinction, and compared to other consonants, consonant minimal pairs /p-p^h^, t-t^h^, k-k^h^, p-t, t^h^-k^h^, t-k/ were more difficult to perceive in noise than in quiet.

Discrimination involving place contrasts of alveolar and palato-alveolar sounds (/s-ʂ, ts-tʂ, ts^h^-tʂ^h^/) was particularly more difficult in quiet. However, the discrimination of these consonant minimal pairs was not affected by noise (i.e., discrimination scores obtained in quiet and in noise did not differ significantly). These findings provide further evidence for the robustness of the acoustic information of fricatives and affricates in noise.

## 5. Limitations and future studies

Only ten participants were included for each age group due to the effects of pandemics and the exploratory nature of the study (e.g., determining appropriate S/Ns and suitability of the test). Therefore, the current findings should be considered preliminary, and future studies using a large sample size are needed to verify the findings.

## 6. Summary

In general, a developmental trend of consonant discrimination was observed, and children were able to discriminate most consonant minimal pairs with better performance in quiet than in noise. Regardless of listening conditions, the contrasts of /p^h^-t^h^/, /k^h^-x/, and /k-x/ were found to be difficult in the discrimination task. In addition, place contrasts between alveolar and palato-alveolar consonants were difficult in quiet, and discrimination contrasted by place of articulation was found to be more difficult in quiet compared to contrasts of aspiration, voicing, and manner of articulation. Three findings have not been reported in previous research. That is, 1) velar contrasts were difficult in quiet and in noise; 2) plosives were difficult when masked by noise; and 3) discrimination of affricates and fricatives were less affected by noise.

## 7. Clinical implications

The research into development of consonant perception in typically developing children can provide a useful reference for the rehabilitation of children with HL and for the evaluation of hearing devices [36]. The current study is the first to examine Mandarin consonant discrimination under both quiet and noisy listening conditions. The findings are expected to help researchers and clinicians not only gain a better understanding of consonant discrimination, but also provide a reference for comparison with children with compromised hearing ability. They highlight the need to consider age and listening conditions when examining consonant discrimination. The test materials used in the current study were found to be age-appropriate for most children; therefore, they can be used in clinical contexts on children with HL who are of similar chronological age, with the expectation that children with HL would perform less well.

## Supporting information

S1 File (DOCX)

## Abbreviations

HL: Hearing loss
NH: Normal hearing
SSN: Speech-spectrum shaped noise maskers
