# Supplementary material for: Discrimination of consonants in quiet and in noise in Mandarin-speaking children with normal hearing
Source: PLoS One. 2023 Mar 21;18(3):e0283198. doi: 10.1371/journal.pone.0283198 (PMC10030016; doi:10.1371/journal.pone.0283198)
Supplement: S1 File — (DOCX) [file pone.0283198.s001.docx]

**Appendix 1**

**33 Mandarin Consonant Minimal Pairs and Test Stimuli**

| Contrastive features | Test Stimuli | | |
| --- | --- | --- | --- |
|  | Pinyin | IPA | Chinese |
| Aspiration | b-p | p-p^h^ | 白-牌 |
|  | d-t | t-t^h^ | 打-塔 |
|  | g-k | k-k^h^ | 狗-口 |
|  | z-c | ts-ts^h^ | 枣-草 |
|  | zh-ch | tʂ-tʂ^h^ | 纸-尺 |
|  | j-q | tɕ-tɕʰ | 鸡-七 |
| Manner of articulation | d-z | t-ts | 岛-枣 |
|  | d-s | t-s | 岛-扫 |
|  | s-c | s-ts^h^ | 四-刺 |
|  | s-z | s-ts | 四-字 |
|  | t-s | t^h^-s | 毯-伞 |
|  | t-c | t^h^-ts^h^ | 兔-醋 |
|  | n-l | n-l | 泥-梨 |
|  | sh-zh | ʂ-tʂ | 书-猪 |
|  | sh-ch | ʂ -tʂ^h^ | 手-丑 |
|  | x-q | ɕ-tɕʰ | 笑-翘 |
|  | x-j | ɕ-tɕ | 写-姐 |
|  | g-h | k-x | 瓜-花 |
|  | k-h | k^h^-x | 苦-虎 |
| Place of articulation | b-d | p-t | 包-刀 |
|  | d-g | t-k | 刀-高 |
|  | p-t | p^h^-t^h^ | 胖-烫 |
|  | t-k | t^h^-k^h^ | 兔-裤 |
|  | m-n | m-n | 买-奶 |
|  | f-s | f-s | 翻-三 |
|  | s-sh | s-ʂ | 三-山 |
|  | sh-x | ʂ-ɕ | 拴-宣 |
|  | x-h | ɕ-x | 选-缓 |
|  | z-zh | ts-tʂ | 足-竹 |
|  | zh-j | tʂ-tɕ | 转-卷 |
|  | c-ch | ts^h^-tʂ^h^ | 草-吵 |
|  | ch-q | tʂ^h^-tɕ^h^ | 穿-圈 |
| Voicing | sh-r | ʂ- ʐ | 射-热 |

**Appendix 2**

Mean Discrimination Scores (%) of Minimal Pairs in Quiet

| Contrastive pairs | Adults | P2 | P1 | K3 | K2 |
| --- | --- | --- | --- | --- | --- |
| ɕ-tɕ | 100.00 | 95.00 | 100.00 | 100.00 | 97.50 |
| ts-ts^h^ | 100.00 | 100.00 | 100.00 | 100.00 | 97.50 |
| tʂ^h^-tɕ^h^ | 100.00 | 100.00 | 95.00 | 100.00 | 97.50 |
| t-t^h^ | 100.00 | 100.00 | 97.50 | 100.00 | 97.50 |
| th-s | 100.00 | 97.50 | 97.50 | 95.00 | 95.00 |
| t-s | 100.00 | 97.50 | 100.00 | 97.50 | 95.00 |
| s-ts | 100.00 | 97.50 | 97.50 | 100.00 | 95.00 |
| t-ts | 100.00 | 97.50 | 100.00 | 100.00 | 95.00 |
| k-k^h^ | 97.50 | 97.50 | 95.00 | 97.50 | 92.50 |
| ɕ-x | 100.00 | 100.00 | 95.00 | 100.00 | 90.00 |
| tʂ-tɕ | 100.00 | 95.00 | 100.00 | 100.00 | 90.00 |
| k-x | 100.00 | 100.00 | 95.00 | 95.00 | 90.00 |
| ʂ-tʂ | 100.00 | 97.50 | 95.00 | 95.00 | 90.00 |
| p-p^h^ | 100.00 | 100.00 | 100.00 | 90.00 | 87.50 |
| f-s | 100.00 | 97.50 | 95.00 | 95.00 | 85.00 |
| ʂ-ɕ | 97.50 | 100.00 | 100.00 | 100.00 | 85.00 |
| m-n | 97.50 | 100.00 | 100.00 | 92.50 | 85.00 |
| ʂ-tʂ^h^ | 100.00 | 100.00 | 100.00 | 97.50 | 85.00 |
| t^h^-k^h^ | 100.00 | 100.00 | 95.00 | 92.50 | 85.00 |
| tʂ-tʂ^h^ | 100.00 | 90.00 | 95.00 | 100.00 | 82.50 |
| ts^h^-tʂ^h^ | 100.00 | 100.00 | 95.00 | 92.50 | 82.50 |
| t-k | 100.00 | 92.50 | 97.50 | 90.00 | 82.50 |
| tɕ-tɕʰ | 100.00 | 100.00 | 100.00 | 97.50 | 80.00 |
| ʂ-ʐ | 100.00 | 97.50 | 100.00 | 97.50 | 80.00 |
| ɕ-tɕʰ | 100.00 | 100.00 | 97.50 | 100.00 | 80.00 |
| s-ts^h^ | 97.50 | 97.50 | 97.50 | 85.00 | 80.00 |
| s-ʂ | 97.50 | 95.00 | 100.00 | 87.50 | 72.50 |
| p^h^-t^h^ | 95.00 | 75.00 | 75.00 | 67.50 | 70.00 |
| p-t | 100.00 | 97.50 | 97.50 | 75.00 | 67.50 |
| t^h^-ts^h^ | 100.00 | 97.50 | 95.00 | 72.50 | 65.00 |
| n-l | 100.00 | 97.50 | 97.50 | 80.25 | 65.00 |
| ts-tʂ | 95.00 | 87.50 | 90.00 | 67.50 | 57.50 |
| k^h^-x | 100.00 | 95.00 | 95.00 | 80.00 | 57.50 |

*Note:* Contrastive pairs in the shaded areas are those pairs scored more than ± 1 standard deviation (SD) from the overall mean of the particular age group. The order is arranged according to performance in K2 kids in quiet**.**

**Appendix 3**

*Mean Discrimination Scores (%) of Contrastive Pairs in Noise Averaged from 0 dB and -5 dB S/N*

| Contrastive pairs | Adults | P2 | P1 | K3 | K2 |
| --- | --- | --- | --- | --- | --- |
| tɕ-tɕʰ | 100.00 | 97.50 | 96.25 | 92.50 | 85.00 |
| ɕ-x | 95.00 | 91.25 | 95.00 | 87.50 | 82.50 |
| ɕ-tɕ | 100.00 | 95.00 | 92.50 | 91.25 | 80.00 |
| tʂ-tʂ^h^ | 98.75 | 96.25 | 92.50 | 90.00 | 78.75 |
| f-s | 98.75 | 91.25 | 96.25 | 78.75 | 78.75 |
| tʂ-tɕ | 97.50 | 93.75 | 93.75 | 88.75 | 78.75 |
| ʂ-ɕ | 98.75 | 95.00 | 90.00 | 86.25 | 77.50 |
| m-n | 92.50 | 82.50 | 76.25 | 81.25 | 76.25 |
| ʂ-tʂ^h^ | 98.75 | 85.00 | 92.50 | 91.25 | 75.00 |
| ts-ts^h^ | 93.75 | 80.00 | 81.25 | 82.50 | 75.00 |
| t^h^-s | 92.50 | 86.25 | 81.25 | 88.75 | 75.00 |
| tʂ^h^-tɕ^h^ | 95.00 | 96.25 | 88.75 | 91.25 | 73.75 |
| t-s | 95.00 | 82.50 | 85.00 | 70.00 | 72.50 |
| ʂ-ʐ | 92.50 | 86.25 | 88.75 | 83.75 | 71.25 |
| s-ts | 93.75 | 95.00 | 93.75 | 87.50 | 70.00 |
| ts^h^-tʂ^h^ | 97.50 | 85.00 | 90.00 | 75.00 | 70.00 |
| t^h^-ts^h^ | 93.75 | 83.75 | 90.00 | 77.50 | 70.00 |
| ɕ-tɕʰ | 95.00 | 96.25 | 92.50 | 86.25 | 68.75 |
| k-k^h^ | 86.25 | 67.50 | 78.75 | 71.25 | 65.00 |
| s-ʂ | 100.00 | 96.25 | 93.75 | 88.75 | 62.50 |
| p-t | 81.25 | 73.75 | 78.75 | 72.50 | 62.50 |
| k-x | 48.75 | 61.25 | 56.25 | 51.25 | 62.50 |
| n-l | 93.75 | 61.25 | 73.75 | 72.50 | 62.50 |
| t-ts | 88.75 | 76.25 | 71.25 | 70.00 | 57.50 |
| t-k | 62.50 | 47.50 | 57.50 | 56.25 | 56.25 |
| ʂ-tʂ | 90.00 | 77.50 | 82.50 | 81.25 | 55.00 |
| t-t^h^ | 85.00 | 76.25 | 73.75 | 73.75 | 55.00 |
| t^h^-k^h^ | 61.25 | 53.75 | 53.75 | 53.75 | 53.75 |
| ts-tʂ | 92.50 | 81.25 | 75.00 | 65.00 | 52.50 |
| s-ts^h^ | 85.00 | 71.25 | 73.75 | 65.00 | 52.50 |
| p-p^h^ | 65.00 | 55.00 | 57.50 | 55.00 | 52.50 |
| k^h^-x | 55.00 | 52.50 | 46.25 | 51.25 | 51.25 |
| p^h^-t^h^ | 50.00 | 47.50 | 52.50 | 46.25 | 47.50 |

*Note:* Contrastive pairs in the shaded areas are those pairs scored more than ± 1 standard deviation (SD) from the overall mean of the particular age group. The order was arranged according to performance in K2 kids in noise.
